# Self-Blinking Thioflavin T for Super-resolution Imaging

**DOI:** 10.1021/acs.jpclett.4c00195

**Published:** 2024-07-19

**Authors:** Qiqi Yang, Elnaz Hosseini, Peigen Yao, Sabine Pütz, Márton Gelléri, Mischa Bonn, Sapun H. Parekh, Xiaomin Liu

**Affiliations:** † Max Planck Institute for Polymer Research, Ackermannweg 10, 55128 Mainz, Germany; ‡ Institute of Molecular Biology gGmbH, Ackermannweg 4, 55128 Mainz, Germany; § Department of Biomedical Engineering, University of Texas at Austin, Austin, Texas 78712, United States

## Abstract

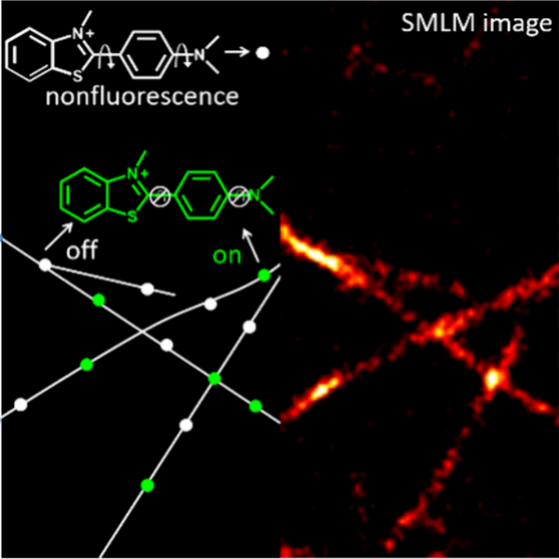

Thioflavin T (ThT) is a typical dye used to visualize the aggregation and formation of fibrillar structures, e.g., amyloid fibrils and peptide nanofibrils. ThT has been considered to produce stable fluorescence when interacting with aggregated proteins. For single-molecule localization microscopy (SMLM)-based optical super-resolution imaging, a photoswitching/blinking fluorescence property is required. Here we demonstrate that, in contrast to previous reports, ThT exhibits intrinsic stochastic blinking properties, without the need for blinking imaging buffer, in stable binding conditions. The blinking properties (photon number, blinking time, and on–off duty cycle) of ThT at the single-molecule level (for ultralow concentrations) were investigated under different conditions. As a proof of concept, we performed SMLM imaging of ThT-labeled α-synuclein fibrils measured in air and PBS buffer.

Amyloid and amyloid-like fibrils (e.g., peptide nanofibrils), formed from proteins and peptides, have emerged as important topics in recent years for various scientific endeavors in physics,^1,2^ chemistry,^3,4^ biology,^5,6^ and medicine.^7,8^ While some functional amyloids with beneficial biological activities have been studied,^9,10^ disease-associated amyloid fibrils remain the focus of much current research. For example, neurodegenerative diseases are age-related diseases for which pathophysiology is diverse, such as memory and cognitive impairments and reduced ability to move, speak, and breathe.^11,12^ Alzheimer’s, Parkinson’s, and Huntington’s disease, all common neurodegenerative diseases, are associated with the misfolding of protein monomers and the formation of aggregated fibrous deposits in the brain.^13,14^ α-Synuclein (α-Syn), a key protein implicated in Parkinson’s disease pathology, exhibits remarkable conformational plasticity, as it can adopt a wide range of structural conformations (oligomers, protofibrils, and fibrils).^15,16^ Each α-Syn conformation displays distinct properties in terms of neurotoxicity, stability, and seeding and propagation ability.^17^ Therefore, detection of different conformations and a mechanistic understanding of the aggregation kinetics and the structural properties of α-Syn are of diagnostic importance and have therapeutic implications.

To visualize the protein aggregation and amyloid formation, many microscopy techniques have been applied, e.g., electron microscopy (EM),^18^ atomic force microscopy (AFM),^19^ and fluorescence microscopy.^20^ Compared with EM and AFM, fluorescence microscopy has the advantages of simple sample preparation, high contrast and excellent compatibility with biological samples. The development of optical super-resolution nanoscale imaging techniques, going beyond the diffraction limit of conventional light microscopy, has further enhanced the potential of fluorescence imaging in recent years.^21−25^ Among the developed optical super-resolution techniques, single-molecule localization microscopy (SMLM), which typically achieves super-resolution by localizing individual molecules, is a popular method.^26,27^

Thioflavin T (ThT), a commonly used fluorescent dye for studies of amyloid fibril formation,^28^ is weakly fluorescent in solution due to internal rotation of the aromatic structure that is associated with intramolecular charge-transfer processes,^29,30^ but it exhibits considerably enhanced fluorescence when bound to amyloid fibrils. Recently, researchers have done work using ThT for the visualization of amyloid fibrils with the SMLM method.^31−33^ Similar to the points accumulation for imaging in nanoscale topography (PAINT) technique,^25^ ThT molecules bind to fibrils and display fluorescence that is essentially absent in the unbound state. This on–off switching of the fluorescent signal is caused by transient binding and unbinding, which creates a type of fluorescent blinking that enables single-molecule localization.^31,32,34^ However, for PAINT, it is important to consider the probe–target affinity (association and dissociation) to ensure that it is in the right range to achieve effective PAINT conditions.^35^ For many stably bound labels, the on–off behavior of organic dyes is usually controlled by chemical oxidation or reduction, called a reducing and oxidizing system (ROXS), depending on the possibility of inducing efficient electron transfer in the system.^36,37^ Accordingly, enhanced by proper buffer conditions, the relaxation of ThT to the twisted state, its intersystem crossing to triplet state, and its potential electron affinity in the twisted state make ROXS likely a favorable route for dark-state control.^33^ However, the specific mechanism has not been studied, and different types of reducing agents in buffer solution have a significant influence on the performance of the blinking.^33^

In this work, we demonstrate that instead of transient binding or buffer-assisted blinking, ThT can act as a self-blinking fluorophore for super-resolution imaging when stably bound to α-Syn amyloid fibrils. We found that unbound ThT molecules showed consistent blinking behavior when coated on a coverslip and embedded in polystyrene (PS) film as well as bound to fibrils. Based on single-molecule analysis, ThT shows blinking properties comparable to those of the current gold standard organic molecules, which, however, require specific blinking buffer conditions. We have demonstrated that ThT showed blinking in different environments (air, polymer film, PBS, and also different buffer conditions), which suggested that the blinking mechanism of ThT is distinct from former reported works, including PAINT (transient binding/unbinding)^32^ and typical ROXS.^33^ Furthermore, as a proof-of-principle demonstration that ThT can be used for super-resolution imaging applications, we imaged α-Syn fibrils labeled with ThT in both air and PBS buffer conditions.

To investigate the photophysical properties of ThT at the single-molecule (ultralow-concentration) level, we first prepared ThT embedded in a PS film under a nitrogen environment. We hypothesized that in this case the rotation of the molecules would be restricted and the nitrogen environment might considerably reduce the possible photobleaching by oxygen (see the Supporting Information (SI) for more information). Using wide-field super-resolution microscopy with 488 nm excitation (see the SI for additional details), instead of stable fluorescence, ThT embedded in PS film showed intrinsic stochastic blinking. As shown in Figure 1a,b, compared with the first frame of the wide-field image, the reconstruction (time projection) image of 20,000 frames of the same imaging area shows a much higher count (number of fluorescent spots) and higher fluorescence intensity. Comparing Figure 1a and Figure 1b, we note that only 22% of the points were detected in the first frame, namely, 197 (0.12 molecule/μm^2^) out of 894 (0.56 molecule/μm^2^), indicating that only 22% of ThT molecules were in the fluorescence-on state after excitation, which conflicts with common understanding. The proportion of detected points in the first frame obtained from three sets of measurements was 20% ± 3% (Table S1). Intuition and previous work suggest that the molecules should stay in the fluorescence-on state once excited and usually need a much stronger laser intensity to push them into the fluorescence-off state.^38^ We note here that the samples were prepared in normal room light condition. For ThT molecules, our hypothesis is that the fluorescence-off state might be induced by excitation of room light during the sample preparation or the weak laser intensity used to find the imaging focal plane. As a test for this hypothesis, additional ThT in PS film samples were prepared in the dark, and up to 56% of the ThT molecules were detected in the first frame, consistent with our hypothesis that room lights seem to push ThT in the PS film into the dark state (see Figure S1 for more information). Moreover, both observations suggest that the fluorescence-on state of ThT molecules can be achieved under continuous laser excitation imaging conditions without the need of the blinking buffer. Meanwhile, the control experiment, fluorescence detection of pure PS film (Figure S2), showed that fluorescent impurities had little impact on the ThT experimental results, even though there were some weakly detectable fluorescent impurities. Furthermore, similar blinking phenomena were observed for ThT molecules immobilized directly on coverslips under nitrogen and normal air conditions in the absence of PS (Figures S3–S5). These observations indicate that oxygen has a negligible effect on the blinking properties of ThT molecules.

**Figure 1:**
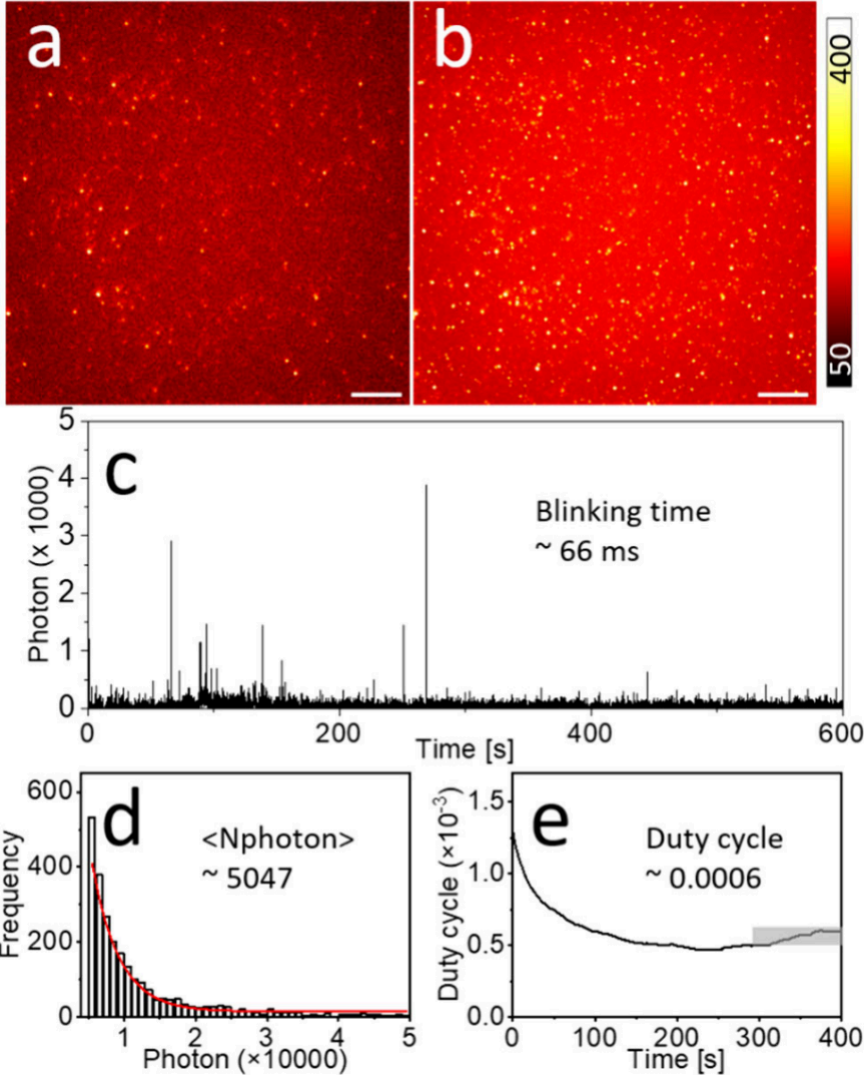
Photophysical properties of ThT embedded in PS film. (a) First frame of wide-field image and (b) reconstruction (time projection) image of 20,000 frames. (c) Time trace of a typical imaging position of one molecule. (d) Histogram of detected photons per switching event and its single-exponential fit. The mean photon number ⟨Nphoton⟩ was determined by the exponential fit. (e) On–off duty cycle (fraction of time a molecule resides in its fluorescent state) of ThT calculated from single-molecule fluorescence time traces. The equilibrium duty cycle was calculated within the time window of 300–400 s (gray box). Scale bar: 5 μm. Color bar: photons per pixel (pixel size of 100 nm).

The blinking properties of ThT at the single-molecule level (at ultralow concentration) were further quantitatively analyzed. For optical super-resolution SMLM imaging, blinking fluorescence with high photon numbers (detected photons per switching event) and low on–off duty cycle (fraction of time a molecule resides in its fluorescent state) are preferred. High photon numbers provide high imaging resolution, while a low on–off duty cycle improves both the imaging accuracy and labeling density by decreasing the probability of more than one fluorophore fluorescing simultaneously within a diffraction-limited area. As shown by the representative time trace of one typical imaging position in Figure 1c, ThT exhibits intrinsic stochastic blinking with an average blinking time of 66 ms per switching event in the PS film (Figure S6). Representative wide-field images from individual molecules corresponding to time trace (Figure S7) and movie (Movie S1) show the blinking of ThT molecules. Figure 1d shows the histogram of detected photons per switching event, and mean photon numbers, determined by the exponential fit, larger than 5000 were obtained. The on–off duty cycle of 6 × 10^–4^ for ThT was calculated from single-molecule fluorescence time traces (Figure 1e). Again, ThT prepared on glass coverslips under nitrogen or in air exhibits similar blinking properties as ThT embedded in PS film (Figures S4 and S5). Using the same sample preparation and data analysis method as in previous publications,^39,40^ ThT shows comparable blinking properties as the current gold standard organic molecules (e.g., Alexa 647), which, however, need the specific blinking buffer condition. Furthermore, we have compared the blinking properties in environments with different amounts of oxygen (samples exposed in air, in a polymer film, or sealed in a N_2_ chamber and samples mounted in PBS solution) (Figure S8 and Table S1). From the summarized results, the blinking properties vary in different environments, but the impact on the SMLM imaging quality is negligible, as shown below.

To further assess the potential of ThT for imaging applications, α-Syn fibrils labeled with ThT were prepared, and super-resolution SMLM images were obtained in both air (Figure 2a–e) and PBS buffer (Figure 2f–j) conditions. Figure 2a shows a comparison of wide-field fluorescence and reconstructed SMLM images of α-Syn fibrils measured in air. Figure 2b,c shows wide-field fluorescence and SMLM images, respectively, of the magnified region of interest (ROI) (ROI 1 in Figure 2a). Compared with the wide-field image, the SMLM image shows a much clearer structure of α-Syn fibrils with a calculated average localization precision of 18 nm. Another magnified ROI SMLM image (ROI 2) is shown in Figure 2d, and the corresponding α-Syn fibril line profile indicates a significant enhancement in the resolution of the SMLM image, with an ∼7-fold reduction in the measured fibril width (Figure 2e).

**Figure 2:**
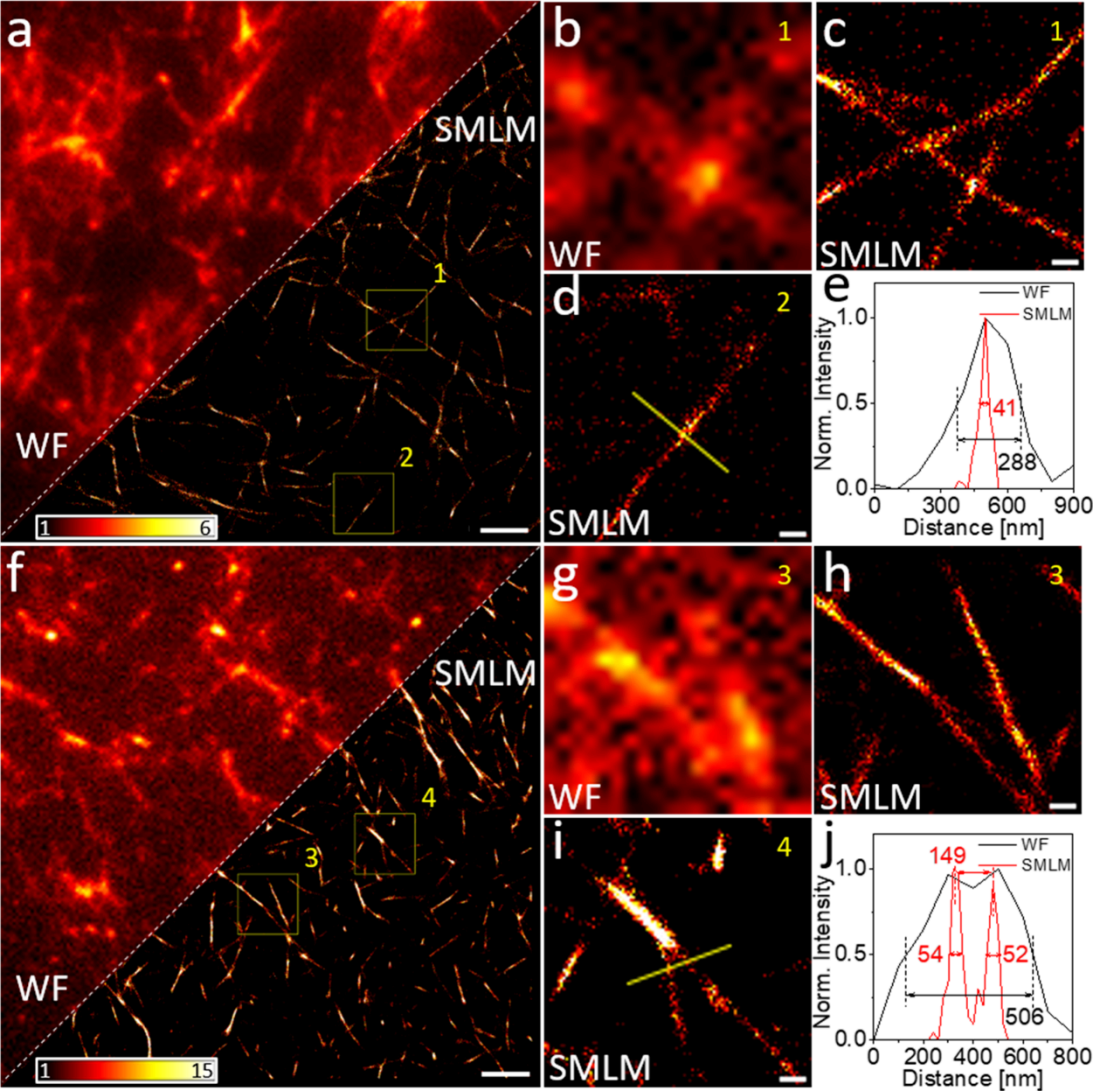
Wide-field (WF) and reconstructed SMLM images of α-Syn fibrils labeled with ThT measured in (a–d) air and (f–i) PBS. (a, f) Comparisons of WF and reconstructed SMLM images. (b, c) and (g, h) Magnified images from region of interest (ROI) 1 in (a) and ROI 3 in (f). (d, i) Magnified images from ROI 2 in (a) and ROI 4 in (f). (e, j) Corresponding line profiles of (d) and (i). Scale bars: (a, f) 2 μm and (c, d, h, i) 200 nm. Color bars: localizations per bin (20 nm × 20 nm).

Similar images of α-Syn fibrils labeled with ThT were also obtained in normal PBS buffer. The α-Syn fibrils were prepared on a coverslip, adhered to the glass surface after drying, and washed, and a PBS solution was dropped on the surface before the measurement (see the SI for more information on sample preparation). Figure 2f shows the comparison of wide-field and reconstructed SMLM images. Similar to the fibrils measured in air, SMLM images of α-Syn fibrils measured in PBS also exhibited better spatial resolution with a calculated mean localization accuracy of 17 nm. The average structural resolution measured by Fourier ring correlation (FRC) is shown in Figure S9, which shows a resolution map of the full image. As shown in the SMLM image of Figure 2i and the corresponding line profiles in Figure 2j, the enhanced SMLM resolution reveals two closely spaced fibrils that are otherwise not distinguishable in a normal wide-field image. The ThT labeling density (number of localizations/length of fibril) is shown in Figure S10. We note here that all measured fluorescence is indeed from ThT, which was confirmed by control experiments showing negligible fluorescent signal when imaging fibrils without ThT labeling (Figure S11).

Compared to those immobilized in PS polymer film (Figure 1) or directly prepared on a coverslip (Figure S4 and S5), lower detected photon numbers were measured for the ThT molecules bound to α-Syn fibrils (Figure 3a,c). This might be due to the different orientation distributions of ThT molecules prepared under different conditions. When immobilized in a thin PS film or directly on a coverslip, ThT molecules may have a tendency to spread more flatly on the coverslip surface.^41^ While bound to amyloid fibrils, ThT is sterically and electronically stabilized by aromatic interactions with close β-sheet interfaces and tends to be in an orientation parallel to the fibrils.^42,43^ Meanwhile, during imaging, a circularly polarized laser beam was used, thus providing only transverse polarization parallel to the imaging plane. As a consequence, part of ThT molecules aligned on fibrils with the tendency in the axial orientation direction have less dipole–dipole coupling for light–matter interaction, resulting in a lower detected average fluorescence photon number.^33,41,44,45^

**Figure 3:**
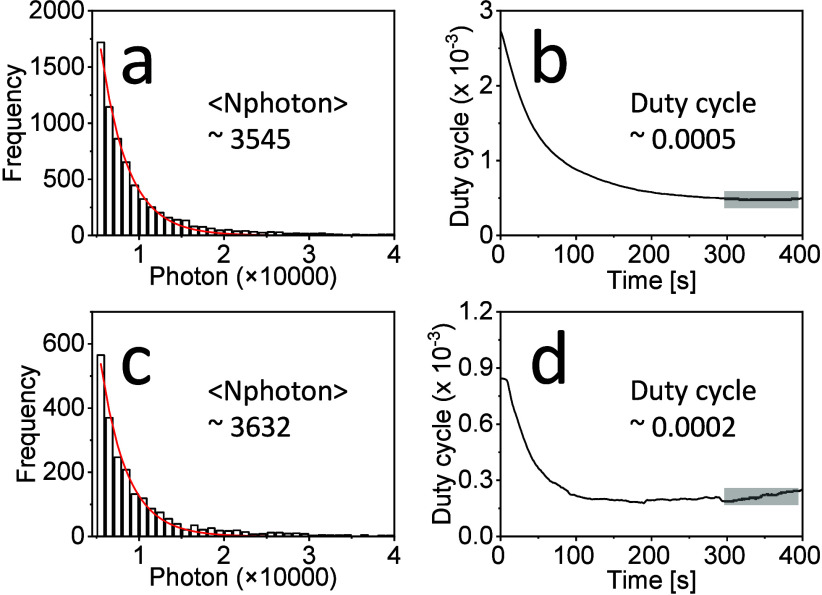
Blinking properties of ThT labeled on α-Syn fibrils measured (a, b) in air and (c, d) in PBS buffer. (a, c) Histograms of detected photons per switching event and single-exponential fits. (b, d) On–off duty cycles of ThT calculated from single-molecule fluorescence time traces.

When ThT molecules were immobilized on fibrils, ThT showed similar blinking properties (photon number, duty cycle, blinking time) when imaged in air and PBS (Figures 3 and S12). We note here that for imaging in PBS conditions, proper sample preparation is crucial. As shown in Figure S13, different blinking properties were characterized when the fibrils were not properly washed and there were free ThT molecular residues. In this case, the detected photon number is lower. Also, the average blinking time of ThT on unwashed fibrils measured in PBS buffer was a bit shorter than that of ThT immobilized on fibrils measured in air and in PBS buffer (33 ms vs 38 and 37 ms; Figures S12 and S13). A possible explanation is that if the fibrils on the coverslip were not washed properly, the molecules remaining on the coverslip and not tightly bound to the fibrils can be dissociated in the solution, making it difficult to avoid blinking signals from transient binding–unbinding. This is because, due to the ∼μM level dissociation constant of ThT to α-Syn fibrils,^46,47^ the transient binding of weakly bound ThT molecules to fibrils is likely and can convolute these measurements. Other work has shown an average blinking time of 12 ms^32^ for transient binding of ThT to amyloid fibrils. This may be the reason for the reduced blinking time measured in PBS buffer compared to air: both types of fluorescent blinking are present when measured in PBS solution. One is the intrinsic blinking of ThT molecules bound to fibrils, which is similar to the one measured in air, and the other is the fluorescence on–off caused by the transient binding–unbinding between ThT molecules and amyloid fibrils. This is also evidenced by the calculated duty cycle (Figure S13), which shows a non-flat but increased duty cycle: more blinking events were detected in this time range around 300–400 s when measured in PBS solution without the proper sample preparation procedure.

Finally, the blinking properties of ThT labeled on amyloid Aβ42 in different buffers were studied. Different blinking properties were characterized (Figure S14 and Table S2), and most notably, different blinking frequencies and duty cycles were observed (Figure S14 and Table S2). On the one hand, this result indicates that oxygen scavengers and reductants do affect the switching between the fluorescent and nonfluorescent states, with a possible ROXS path,^37^ which still requires further study. On the other hand, these measured blinking properties (photon number, blinking time, and duty cycle) do not significantly affect the imaging quality in different buffer conditions. It further confirms that no special imaging buffer conditions are required to perform SMLM imaging with tightly bound ThT. The survival fractions (Figure S15) of ThT molecules measured in different environments were consistent with the time traces, where the presence of reductants resulted in larger survival fractions. Furthermore, the fluorescence of ThT was recoverable by illumination of a 405 nm laser (Figures S16 and S17), which implies that ThT might not be photobleached but instead might enter a long dark state. In addition to photoinduced recovery, fluorescence could be spontaneously recovered through a slow thermodynamic process (Figure S18). Even though it is less efficient than light-induced recovery, this property should benefit potential applications that require repeated/multiple imaging with long intervals (e.g., hundreds of seconds). The recovery process and exact blinking mechanisms of ThT are exciting topics for a future study.

In conclusion, we find ThT, a standard dye for detecting fibril aggregation, shows self-blinking and can be used to visualize fibril structures with a single-molecule localization microscopy (SMLM) method directly. That is, blinking properties are observed in the absence of a blinking buffer, which is commonly used with organic dyes. The blinking properties (photon number, blinking time, and on–off duty cycle) of ThT at the single-molecule level are characterized both on a coverslip and embedded in a PS film. We also demonstrate super-resolution images of ThT-labeled α-Syn fibrils measured in air (PBS buffer) with a localization precision of 18 (17) nm. Our work therefore focuses on three key aspects: the observation and characterization of novel blinking properties; applying these newly discovered properties of ThT molecules to SMLM imaging of amyloid fibrils in different environments, including air and PBS buffer; and environment-independent high-quality imaging. Our approach enables high-quality SMLM imaging without environmental limitations.

In the long term, fluorescent molecules with intrinsic blinking will enable easier visualization of topography in biological samples (e.g., different kinds of amyloid fibrils) at high resolution. Meanwhile, the blinking mechanism investigation of ThT may further provide a basis for searching for and synthesizing more types of intrinsic blinking molecules.^48^ Furthermore, the self-blinking properties of ThT without reliance on blinking buffer may contribute to potential applications in correlative light–electron microscopy (CLEM).^49,50^ Though CLEM has been used to study morphology and dynamics of amyloid nanostructures,^51−54^ studies on combined electron microscopy and super-resolution SMLM are still limited.

## Supporting Information Available

Experimental details, data analysis, and characterization details (PDF)
Movie showing the blinking of ThT molecules (AVI)

## Supplementary Material

jz4c00195_si_001.pdf

jz4c00195_si_002.avi
