# Supplementary material for: Self-Blinking Thioflavin T for Super-resolution Imaging
Source: J Phys Chem Lett. 2024 Jul 19;15(30):7591–6. doi: 10.1021/acs.jpclett.4c00195 (PMC11299178; doi:10.1021/acs.jpclett.4c00195)
Supplement: Supplementary file 1 — jz4c00195_si_001.pdf [file jz4c00195_si_001.pdf]

Supplementary information

Self-blinking Thioflavin T for Super-resolution Imaging

Qiqi Yang[+,a] *Elnaz Hosseini*[+,a] *Peigen Yao*[a] *Sabine Pütz*[a] *Márton Gelléri*[b] *Mischa Bonn*[a] *Sapun H. Parekh*[*a,c] *and Xiaomin Liu*[*a]

[a] Max Planck Institute for Polymer Research, Ackermannweg 10, 55128 Mainz, Germany

[b] Institute of Molecular Biology gGmbH, Ackermannweg 4, 55128 Mainz, Germany

[c] Department of Biomedical Engineering, University of Texas at Austin, Austin, TX 78712, USA

Corresponding Author

* sparekh@utexas.edu (S. Parekh); liuxiaomin@mpip-mainz.mpg.de (X. Liu)

General experimental details

Materials. MICRO 90® concentrated cleaning solution was purchased from International Products Corporation. Toluene was purchased from two manufacturers: Sigma-Aldrich (ACS reagent, ≥ 99.5%) and Acros Organics (anhydrous, ≥99.8%). Chloroform was purchased from Sigma-Aldrich (HPLC grade, ≥99.8%). Tetrahydrofuran (THF) was purchased from Sigma-Aldrich (anhydrous, ≥99.9%). Dulbecco's phosphate-buffered saline (DPBS) was purchased from Thermo Fisher Scientific (no calcium, no magnesium). Ethanol and methanol were purchased from Honeywell Research Chemicals (GC grade, ≥99.8%). Thioflavine T was purchased from Merck KGaA. Polystyrene (PS) was purchased from Aldrich (MW ~280,000), and was purified before use. The coverslips (#1.5, 170 µm) were purchased from ibidi GmbH.

Substrate cleaning. The coverslips were sonicated in 1% Micro 90 alkaline cleaning solution for 15 min. Then the substrates were then rinsed three times with Mili-Q water and finally dried with nitrogen flow. Afterwards, the substrates were cleaned by an oxygen-plasma cleaner (250 W, 5-10 minutes).

Preparation of ThT single molecule samples.

ThT on coverslip. 10 µL of a solution of ThT (10^{-12} M in chloroform) was spin-coated on coverslip with speed at 2,000 rpm for 60 s. The coated coverslip was stored at room temperature for 1h for complete evaporation of the solvent. Spin- coating, drying and coverslip sealing were performed under room light in a nitrogen glove box or in air as stated.

ThT embedded in Polystyrene film. 10 µL of a solution of ThT (10^{-12} M in chloroform, with 0.04mg/mL polystyrene) was spin-coated onto coverslip at 2,000 rpm for 60 s. The coated coverslip was stored at room temperature overnight for complete evaporation of the solvent. Spin-coating, drying and coverslip sealing were performed in a nitrogen glove box. ThT PS films were prepared under room light or in dark. For sample preparation in the dark, only the light in glove box was first turned on in a dark laboratory. When dropping the solution on the coverslip, turn off the light. After spin-coating, the coverslip was stored in a box wrapped with tin foil. When sealing coverslip, for visual operation, light of a cellphone from a distance was used to ensure that as little light as possible can illuminate the sample. The sealed sample was stored in foil wrappers before measurement.

Preparation of ThT labelled α-Syn fibrils.

α-Syn was expressed in Escherichia coli BL21-D3 up to OD_{600} of 0.5 and then induced with 0.5 mM IPTG, post induction grows the cells o/n for 15-18 hours at 20 °C. Harvest the cells at 8000g. Briefly, α-Syn WT plasmid containing E. coli cells were grown in Luria broth (LB) overnight in the presence of 100 µg/mL ampicillin. Upon reaching the OD_{600} of 0.5, the cells were induced to express α-Syn using 1 mM IPTG for 4 h, while the culture flasks were incubated in 37 °C and shaken at 180 rpm. Then, the cells were harvested by centrifugation at 6,000 rpm for 15 min at 4 °C to obtain the cell pellet. Following re-suspension of the cell pellet in lysis buffer (20 mM Tris base pH 8.0), sonication at 50 W (12 minutes, 6sec on 9 sec off,) was performed. Then, the cells suspended in the lysis buffer were placed in boiling water for 10 min followed by centrifugation at 18,000 ×g for 30 min. The supernatant was collected and ammonium sulfate was slowly added until reaching 0.36 g/ml. Following stirring for 30 min at 4 °C, the suspension was centrifuged at 18,000 ×g for 20 min at 4 °C. The pellet was re-suspended in 20 mM Tris buffer (pH 8.0) and loaded onto an anion exchange using Q Sepharose. α-Syn was eluted at 300 mM NaCl and its purity was confirmed by SDS-PAGE. Purified α-Syn was dialyzed against PBS 1X pH7.4 (overnight) @ 4 °C using 3K MWCO membrane and its concentration was determined by measuring its absorbance at 275 nm (ε275 = 5600 M^{-1} cm^{-1}). Purified protein at 467 µM was aliquoted and stored at -80°C.

For fibrillation in Eppendorf tubes: 70µM of α-Syn in PBS pH 7.4 was incubated at 37 °C for 48 hour under continuous shaking at 1000 rpm using a Thermomix.

For SMLM fibril imaging in air: ThT was added to pre-formed fibrils (final concentration of 20 µM for fibrils and 2 nM for ThT) and allowed to incubate for 15 minutes in dark place at room temperature. 10 µL of this solution was added to cleaned coverslips and allowed to dry in ambient, dark conditions.

For SMLM fibril imaging in PBS: Cleaned coverslips were treated with 50 µL polylysine solution (0.1% in H_2O w/v) to render them adhesive. After 30 min, the polylysine was washed off with Milli-Q water, and the cover slip was dried with N_2 flow. 20 µL of the fibrils were dropped onto the treated surface. After air-drying in the dark, the coverslip was washed with Milli-Q water for 3 times and dry in the air room temperature overnight. The sample was covered in PBS, sealed, and stored for imaging in the dark at room temperature.

Single-molecule localization microscopy. The single-molecule localization measurements were performed using a super resolution ground state depletion (SR GSD) microscope (Leica). 488 nm (300 mW) laser was selected for fluorescence reactivation. For the 488 nm laser, the excitation filter (483 nm - 493 nm/400 nm - 410 nm), the dichroic beam splitter (496 nm) and the emission filter (505 nm - 605 nm/449 nm – 451 nm) were used. Laser intensity (5.1 kW/cm^2) was obtained by dividing the laser power (82.2 mW was used, tested under the objective) by the area. The objective lens HCX PL APO 160x 1.43 NA Oil CORR-TIRF was selected for single-molecule measurements and super-resolution imaging. The microscope was equipped with an EMCCD camera (iXonDU-897, Andor). The camera settings were 10 MHz at 14 bit and a pre-amplification of 5.1. For super-resolution imaging, the camera exposure time was set to 30 ms and an EM gain of 100 was used. Please note here that the double bandwidth of the filters/beam splitter were chosen for 405 nm back pumping and in our experiments mentioned in this work, such back pumping was not used.

Data analysis

To obtain the number of points from the series frames images, first of all, time projection (maximum intensity projection) was done with all images, and then each point was localized through the ThunderSTORM-plugin in ImageJ[1]. Fluorescence intensity traces were extracted by first generating a maximum intensity projection of the recorded frames. Fluorescence signals in this projection were localized using the ThunderSTORM. We then calculated the intensity trace for each localization throughout all raw data frames as the total background corrected intensity in a 7 x 7 ROI around the localized coordinates. The local background for every localization in every frame was calculated within a 17 x 17 ROI. Pixel values exceeding 5 times the standard deviation within this ROI were excluded from background calculation as they were considered as fluorescence signal. Calculated total intensities within the ROIs were then plotted for every frame.

To calculate the photoelectrons on a camera pixel for an EMCCD, the equation is:

$$N = \frac{p}{g}(I - b)$$

Where N is the number of photoelectrons, p is the EMCCD sensitivity (in photoelectrons per A/D count), g is the EM gain for EMCCD camera, I is the image intensity (in A/D counts) and b is the baseline level (in A/D counts) from a dark image at the identical camera settings. For camera (iXonDU-897, Andor) used in this work, p is 12.09 while g was set to 100, b is 100.

The photon numbers, blinking time, duty cycle and localization precision were analyzed following the reported method[2]. The analysis needs a two-step process. First, ThunderSTORM plugin[1] in ImageJ was used to localize molecules in every frame of the recorded imaging data. A result table including coordinates of localizations, sigma (standard deviation of the Gaussian fitted on the peak), intensity/photons and uncertainty was given. Localizations were then filtered according to the expected width of the sigma (calculated from diffraction limit, we used range of 75-125 for ThT). Localizations appearing in consecutive frames were then merged. As spatial constraint, we used a maximum distance of 80 nm, a rather large radius was chosen to allow localizations with low photon counts to be still properly merged. After merging, new column called detections can appear, which means number of frames where the peak has been detected and fitted. Second, the exported result was processed with matlab. Photon number and blinking time: The histogram of photons was generated and fitted by a single exponential function ($y=ae^{bx}$) with matlab. For the blinking time, histogram of time (detections x exposure time) was generated and fitted. Reported mean values are derived from the fit. Duty cycle: The on-off duty cycle was calculated within a sliding window of 100 s by dividing the on-time of the time window. The equilibrium duty cycle reported was the mean of the duty cycle value between 300–400 s that molecules not yet photobleached. Localization precision: In ThunderSTORM plugin in ImageJ, individual points were localized and the uncertainty was calculated by maximum-likelihood estimation (MLE) algorithm[3]. The reported localization precision was the mean value of histogram of uncertainty obtained. In general, 2-4 measurements were done per condition.

Supplementary Figures

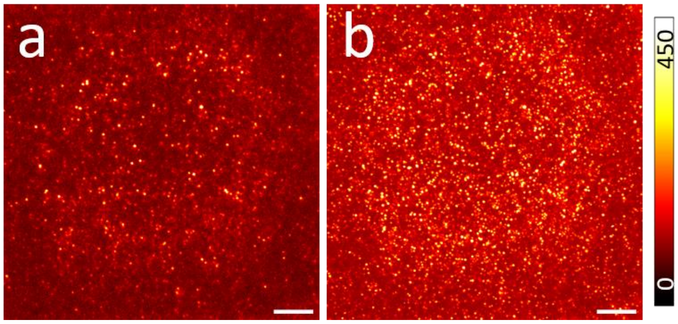

Figure S1**.** Wide-field images of ThT embedded in PS film prepared in a dark environment. (a) The first frame and (b) reconstruction (time projection) image of 20,000 frames. Scale bar: 5 µm. Color bar: photons per pixel (100 nm).

Figures S1a and 1b show the first frame of the wide-field image and the reconstruction (time projection) image of 20,000 frames of the same imaging area of ThT embedded in PS film prepared in dark environment. The sample was prepared on an Ibidi gridded coverslip, with which the focal plane can be easily find under bright field with weak lamp. Then switch to fluorescence mode to collect data. The reconstruction (time projection) image shows more count (number of fluorescent spots) and higher fluorescent intensity. Comparing Figures S1a and 1b, we note that 56% of the spots are detected in the first frame – 910 (0.57 molecules/µm^2) vs. 1635 (1.02 molecules/µm^2), indicating that 56% of ThT molecules are in the fluorescent ON state once excited. Compared with the samples in the Figure 1 (prepared under room light), the samples prepared in the dark show more points are in the fluorescent ON state once excited, indicating that some ThT molecules can be illuminated and pushed into fluorescent OFF state by room light during the sample preparation or the weak laser intensity used to find the imaging focal plane.

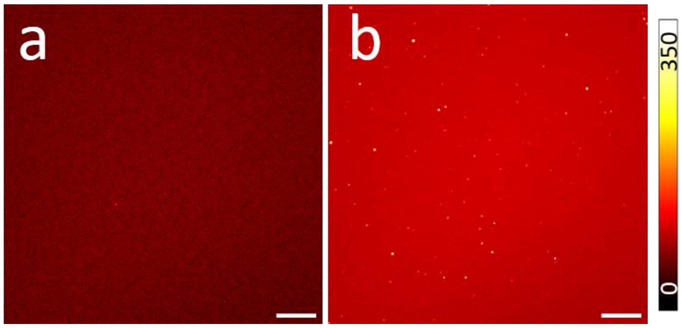

Figure S2**.** Wide-field images of pure PS film. (a) The first frame and (b) time projection image of 20,000 frames. Scale bar: 5 µm. Color bar: photons per pixel (pixel size 100 nm).

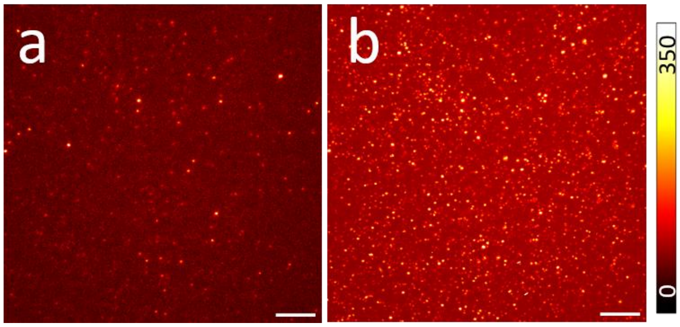

Figure S3. Wide-field images of ThT on coverslip (sealed in N_2) with samples exposed to light. (a) The first frame and (b) time projection image of 20,000 frames. Scale bar: 5 µm. Color bar: photons per pixel (pixel size 100 nm).

Figures S3a and 3b show the first frame of the wide-field image and the reconstruction (time projection) image of 20,000 frames of the same imaging area of ThT prepared on coverslip in nitrogen. The reconstruction (time projection) image shows much more count (number of fluorescent spots) and higher fluorescent intensity. Comparing Figures S3a and 3b, we note that only 22% of the spots are detected in the first frame - 271 (0.17 molecules/µm^2) vs. 1255 (0.78 molecules/µm^2), indicating that only 22% of ThT molecules are in the fluorescent ON state once excited.

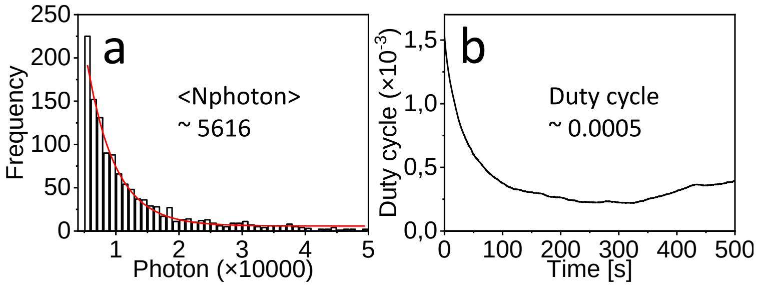

Figure S4. Photophysical properties of ThT on coverslip (sealed in N_2). (a) Histogram of detected photons per switching event and single-exponential fit, mean photon numbers were determined by the exponential fit; (b) on-off duty cycle (fraction of time a molecule resides in its fluorescent state) of ThT calculated from single-molecule fluorescence time trace.

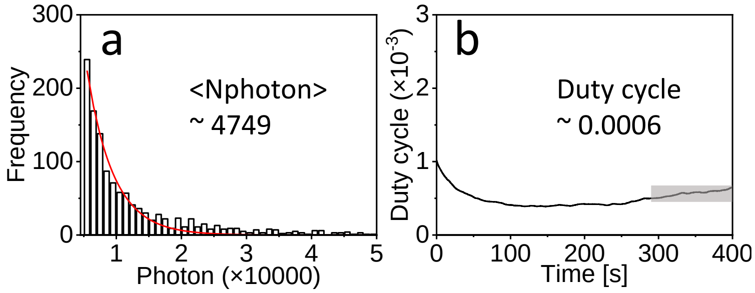

Figure S5**.** Photophysical properties of ThT on coverslip (measured in air). (a) Histogram of detected photons per switching event and single-exponential fit, mean photon numbers were determined by the exponential fit; (b) on-off duty cycle (fraction of time a molecule resides in its fluorescent state) of ThT calculated from single-molecule fluorescence time traces.

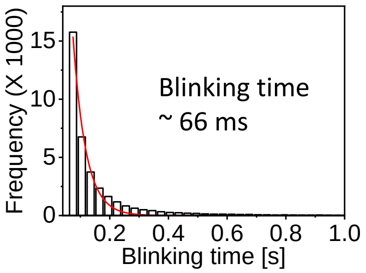

Figure S6**.** Photophysical properties of ThT embedded in PS film (sealed in N_2). Histogram of blinking on time per switching event and single-exponential fit, mean blinking time were determined by the exponential fit.

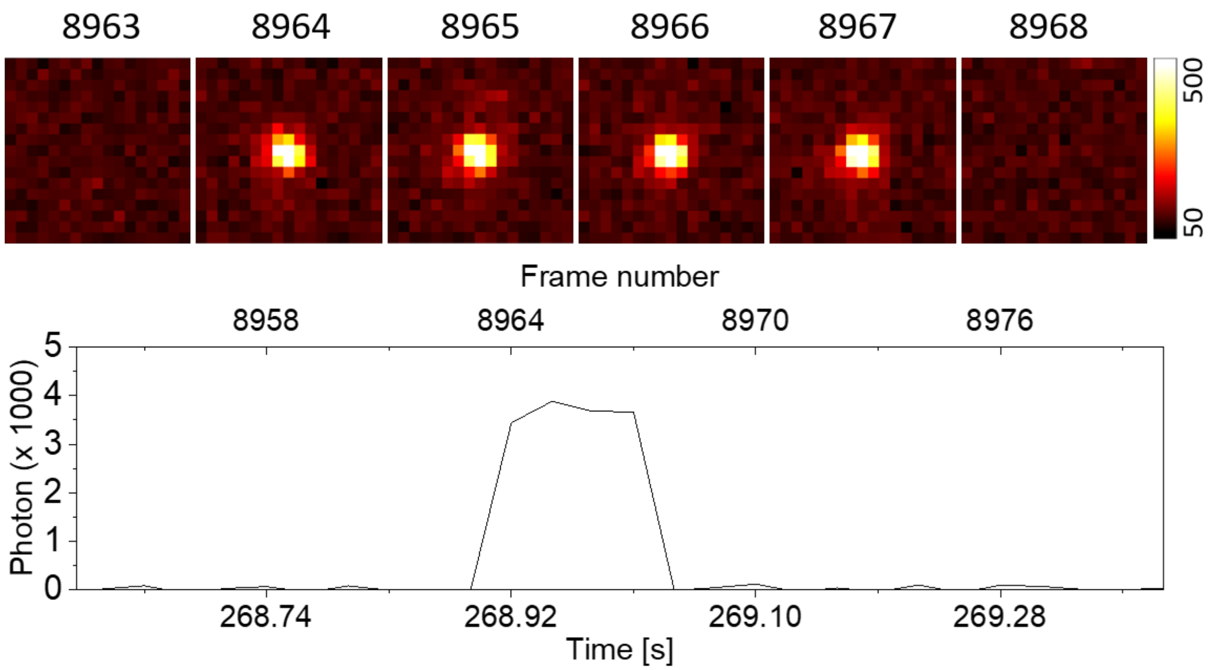

Figure S7. Wide field images at selected frames range from 8963 to 8968 (upper) and corresponding time trace (lower) cut from Fig. 1c. Image size: 17 pixel x 17 pixel. Color bar: photons per pixel (100 nm).

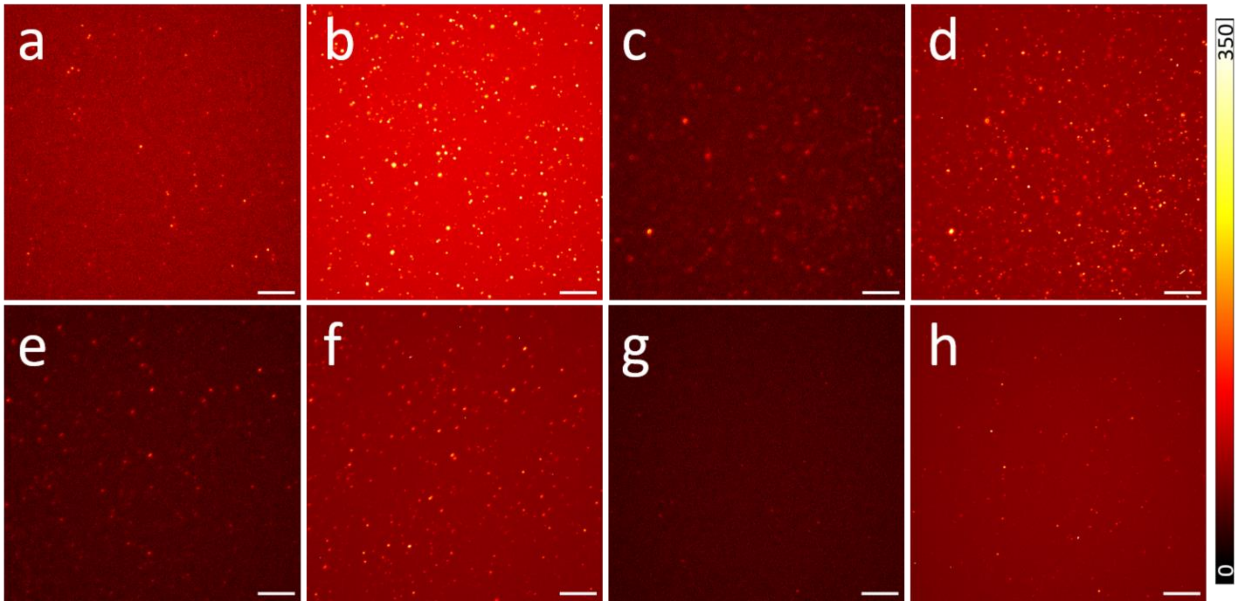

Figure S8. Representative wide-field images of ThT measured in different environments. (a, b) on coverslip measured in air, (c, d) embedded in PS film, (e, f) on coverslip sealed in N_2, (g, h) on coverslip measured in PBS. (a, c, e, g) The first frame and (b, d, f, h) reconstruction (time projection) image of 20,000 frames. Scale bar: 5 µm. Color bar: photons per pixel (pixel size 100 nm). It should be noted here, the samples used in this figure were prepared using the same

protocol as described in ‘‘Preparation of ThT single molecule samples’’, but with ~1/5 concentration of ThT to avoid the possibility of aggregation/overlap of molecules. Comparing the first frame and reconstruction image, the ratio of points detected in the first frame was 22% (114 molecules, 0.07 molecules/μm^2 out of 523 molecules, 0.33 molecules/μm^2), 22% (39 molecules, 0.02 molecules/μm^2 out of 175 molecules, 0.11 molecules/μm^2), 20% (109 molecules, 0.07 molecules/μm^2 out of 543 molecules, 0.34 molecules/μm^2) and 20% (27 molecules, 0.02 molecules/μm^2 out of 135 molecules, 0.08 molecules/μm^2), for environments in air, PS, N2 and PBS, respectively.

It should be noted here, the samples used in this figure were prepared using the same protocol as described in SI ‘‘Preparation of ThT single molecule samples’’, but with ~1/5 concentration of ThT to avoid possibility of aggregation/overlap of molecules. From the summarized results in Table S1 below, the ratio of points detected in the first frame are not affected by the oxygen too much. Meanwhile, the blinking properties (photons and duty cycle) of ThT vary a little bit in different environments, but the impact on SMLM imaging quality is negligible.

Table S1. Summary of blinking properties of ThT measured in different environments.

Environment	PS + N_2[a]	Air[b]	PS[b]	N_2[b]	PBS[c]
Ratio of points detected in the first frame in 20000 frames (%)	20 ± 3	23 ± 5	22 ± 10	21 ± 14	20
Detected photons per switching event	5078 ± 343	5101 ± 316	4885 ± 305	4759 ± 1132	3059
Blinking time [ms]	74 ± 8	67 ± 5	64 ± 7	62 ± 10	66
Duty cycle [x 10^{-4}]	6.0	4.4	5.3	5.0	2.6

[a] ThT embedded in PS film prepared in N_2 glove box, average of 3 measurements. [b] Averaged of 4 measurements. [c] From 1 measurement.

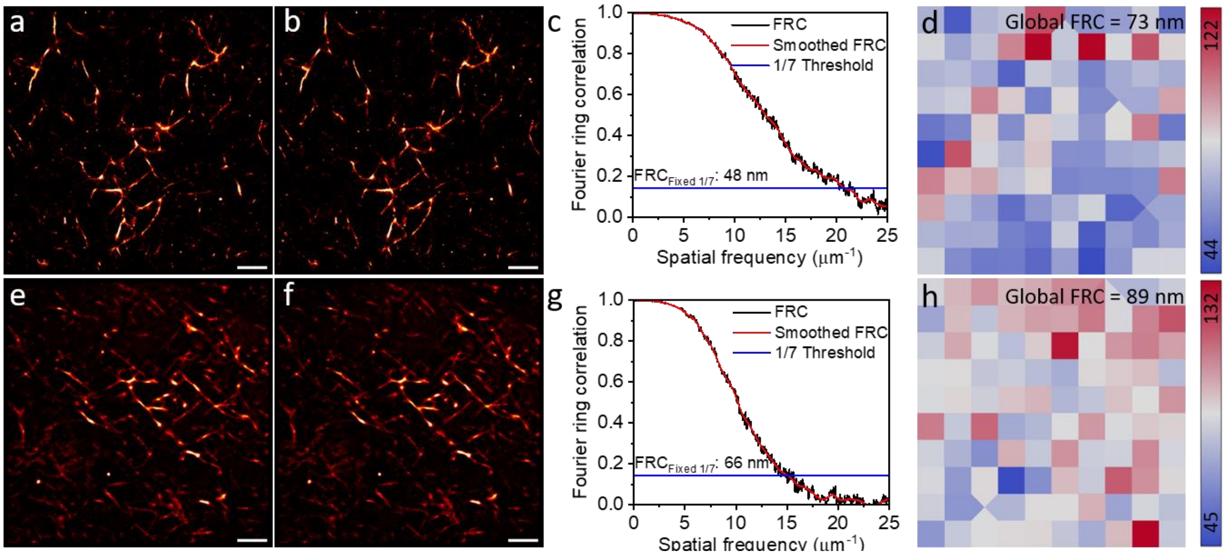

Figure S9. Fourier ring correlation (FRC) analysis for α-Syn fibrils labelled with ThT measured in air (a-d) and PBS (e-h). Odd (a,e) and even (b,f) frames produced independent super-resolution reconstruction images, each from 10000 frames. FRC analysis (c,g) and local mapping of FRC values (d,h) for super-resolution images in a,b and e,f. For the FRC analysis, images were splitted into odd and even frames, which can produce two independent superresolution reconstructions. Within the FRC plugin in ImageJ, the two data reconstructions are divided into blocks and for each block the FRC value is calculated with algorithm described in[4]. NanoJ-SQUIRREL plugin in ImageJ was used to generate FRC mapping[5] and for each block the FRC value is calculated as c and g. The mean FRC values from FRC mapping for ThT labelled on α-Syn fibrils measured in air and PBS are 73 nm (std 16 nm) and 89 nm (std 13 nm), respectively.

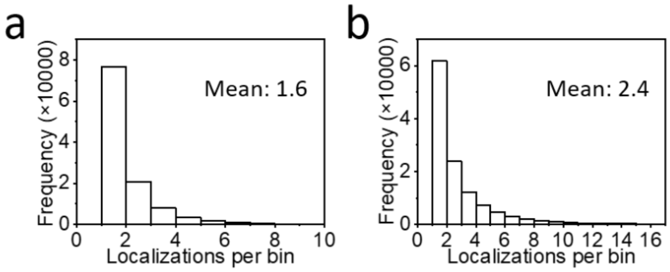

Figure S10. Histogram of the number of localizations per bin (20 × 20 nm^2) corresponding to Figure 2a and f, α-Syn fibrils labelled with ThT measured in air (a) and PBS (b). The number of localizations was given by ThunderSTORM as intensity of image by the visualization mode 'Histogram'. Number of localizations/length of fibril was also estimated. First, 'Apply_DOG_Filtering.py script' from GitHub was used to enhance the image and highlight the strands. Then 'ridge detection' plugin was used to estimate the length of fibrils. Numbers of

localizations/length of fibril of 11.2/μm (measured in air) and 47.5/μm (measured in PBS) were obtained by dividing total number of localizations to total length of fibrils.

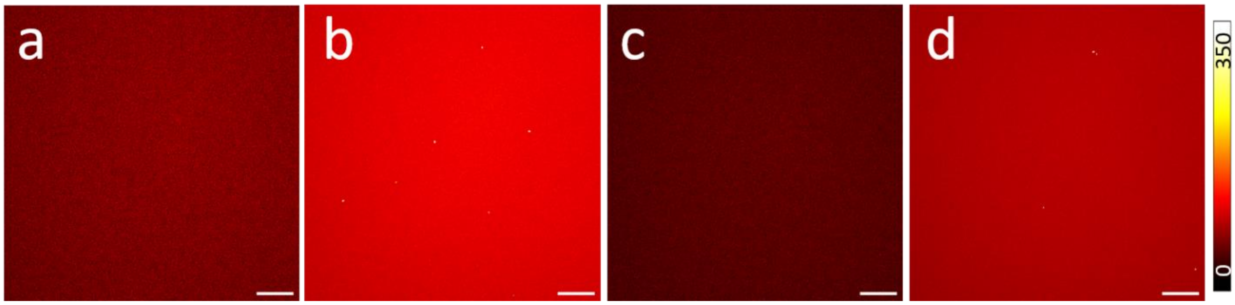

Figure S11**.** Wide-field images of fibrils without dye labelling measured in air (a, b) and in PBS buffer (c, d). (a, c) the first frame and (b, d) reconstruction (time projection) image of 20,000 frames. Scale bar: 5 μm. Color bar: photons per pixel (pixel size 100 nm).

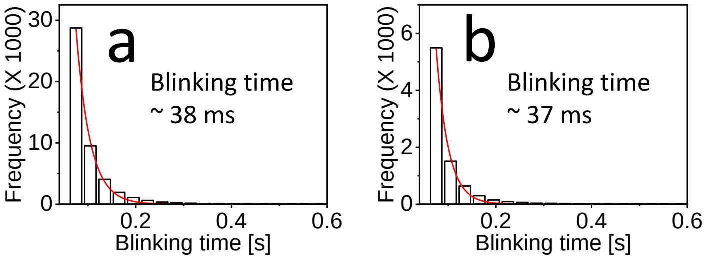

Figure S12**.** Histograms of blinking on time per switching event of ThT that has labelled α-Syn fibrils measured in air (a) and in PBS buffer (b) and single-exponential fit, mean blinking time were determined by the exponential fit.

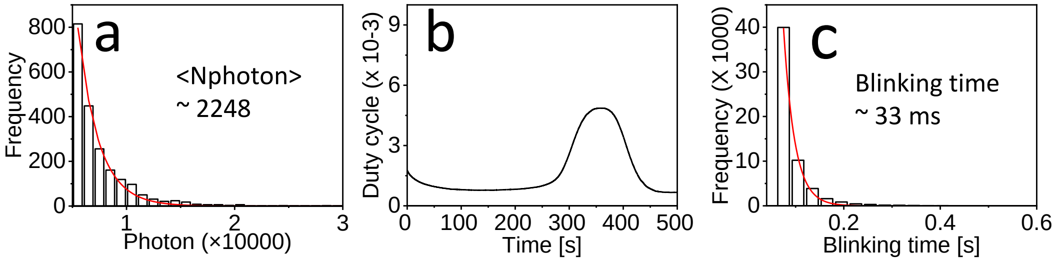

Figure S13**.** Blinking properties of ThT that has labelled α-Syn fibrils measured in PBS buffer where fibrils were not washed properly. (a) Histogram of detected photons per switching event and single-exponential fit. (b) On-off duty cycle of ThT calculated from single-molecule fluorescence time traces; (c) histogram of blinking on time per switching event and single-exponential fit, mean blinking time were determined by the exponential fit. (Compare to Fig. 3 c,d in main text).

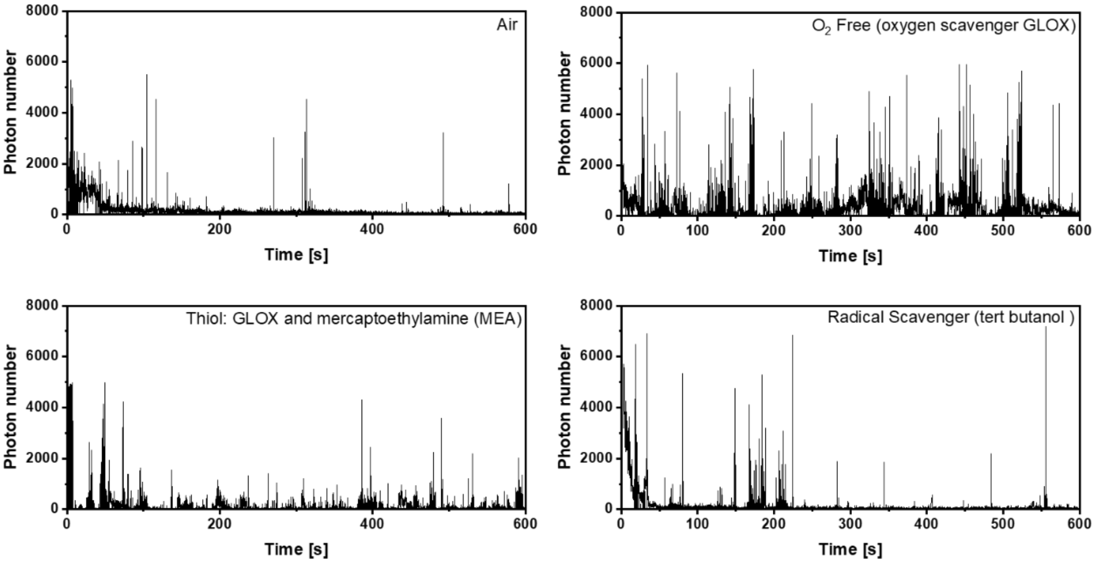

Figure S14**.** Representative time traces of one imaging position of ThT labelled on fibrils measured in different conditions. Here, ThT labelled on amyloid Aβ42 was investigated. Aβ (No. RP10017) were purchased from Genscript as Aβ1–42 "click peptide". These click peptides can be easily converted to native peptide at pH 7.4 or above. Aβ1–42 peptide was diluted with DPBS to a final concentration of 100 μM and stored in a − 80 °C freezer until use. This peptide solution is a mixture of monomers and oligomers since there is no pre-monomerization[6]. For fibril formation, 80 μL DMSO was added to 20 μL Aβ1-42 peptide solution and incubated for 36 hours at 37 °C, and then 1 μL of ThT (2 μM) was added to the fibril solution. After Aβ fibril formation, 10 μL of fibril solution was dropped on a clean circular coverglass (#1.5, 170 μm thickness) and dried in a vacuum desiccator. The fiber-deposited coverglass was washed three times with water to remove excess salt and unfixed fibers and then dried again for further measurement. 10 μL of buffer solution was dropped on the surface for measurement. Air: ambient condition; O2 Free: glucose (1% vol) and glucose oxidase (GLOX, 1% vol) in DPBS; Thiol: glucose, GLOX and mercaptoethylamine (MEA, 10 mM) in DPBS; Radical Scavenger: tert-butanol (10% vol) in DPBS.

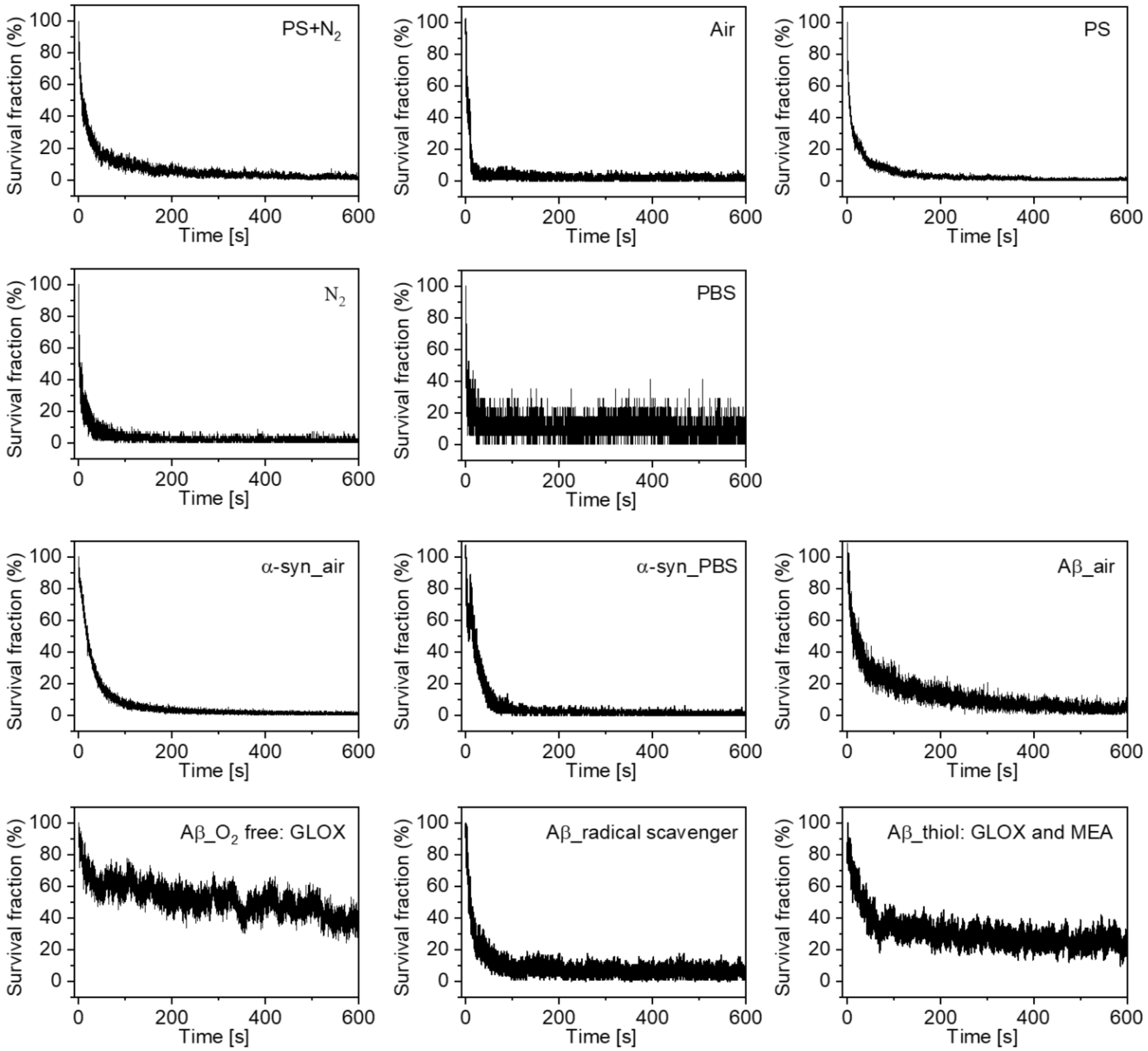

Figure S15. Survival fraction of ThT molecules measured in different environment. The first two rows are from individual ThT molecules (10^{-12} M), while the latter two rows are from ThT molecules on fibrils. A single molecule was defined to be bleached/entering long term fluorescence-off state after its final on-switch. The time-dependent survival fraction was calculated as the number of molecules that are not yet bleached/ entering long term fluorescence-off state divided by the total number of detected molecules. Note that we report the survival fraction for the total imaging time of 600 seconds long.

It is important to note that Dempsey et al.[2] reported the survival fraction over the first 700 seconds out of a total measurement of 2,000 seconds. In our calculations, we calculated the

survival fraction over a total testing duration of only 600 seconds. Many ThT molecules entered deactivation state and were not re-activated in the first 600 seconds, which in our analysis is defined as being photobleached. Therefore, the analytical results as shown in Figure S15 reported a relatively much lower survival fraction as compared with the ones reported in in Dempsey et al.[2]. Consistent with the time traces measured under different buffer conditions shown in Figure S14 of the previous revision, the highest survival fraction was calculated in O_2 Free: glucose (1% vol) and glucose oxidase (GLOX, 1% vol) in DPBS.

Table S2. Summary of blinking properties of ThT measured in different environments.

	Air[a]	Air[b]	O_2 free[b]	Thiol[b]	Radical scavenger[b]
Detected photons per switching event	3545	4174	5400	4435	5975
Blinking time [ms]	38	44	76	79	59
Duty cycle [x 10^{-3}]	0.5	0.8	4.4	1.9	0.5
Localization precision [nm]	18	23	19	22	19

[a] ThT labelled on α-Synuclein; [b] ThT labelled on Aβ42.

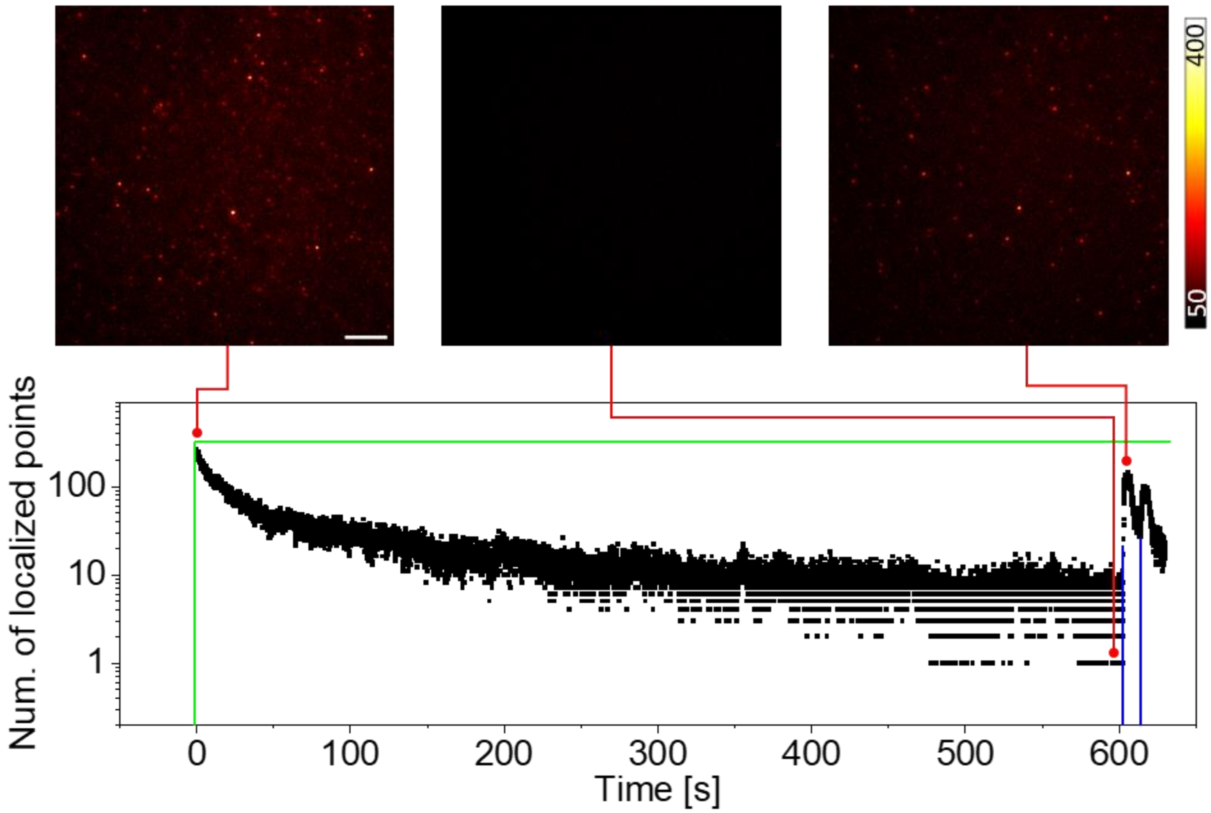

Figure S15. Upper: Representative wide field images at three selected time point as pointed by the red lines; lower: number of detected points per image frame versus the time. The sample (ThT embedded in PS film in N_2) was illuminated continuous with 488 nm laser (5.1 kW cm^{-2}, green line), camera exposure time 30 ms. After about 600 s imaging, an additional 405 nm excitation (0.30 kW cm^{-2}, blue line) was administered and repeated stochastically. Scale bar: 5 μm. Color bar: photons per pixel (100 nm).

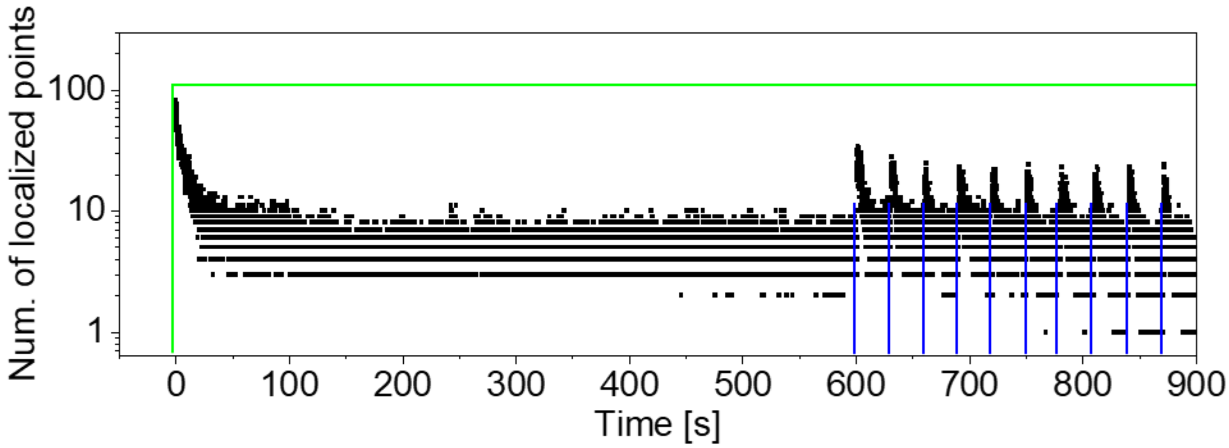

Figure S17. Number of detected points per image frame versus the time. The sample (ThT embedded in PS film in N_2) was illuminated continuous with 488 nm laser (2.23 kW cm^{-2}, green line), camera exposure time 30 ms. After about 600 s imaging, an additional 405 nm excitation (0.30 kW cm^{-2}, blue line) was administered once (~ 1 s) every 30 s and repeated 10 times, and about 42%, 31%, 26%, 27%, 29%, 31%, 18%, 25%, 22%, and 19% of molecules were detected compared with the first frame, respectively.

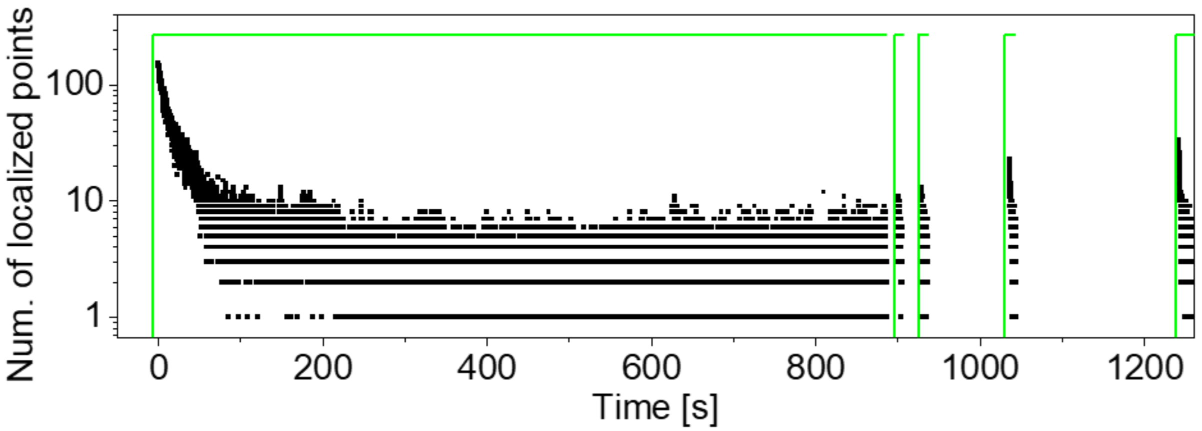

Figure S18. Number of detected points per image frame versus the time. The sample (ThT embedded in PS film in N_2) was illuminated with 488 nm laser (2.23 kW cm^{-2}, green line), camera exposure time 30 ms. After about 900 s continuous imaging, 488 nm laser was turned off for a period of time (duration of 14 s, 23 s, 99 s and 197 s) and then was turned on for imaging again (imaging time of 5 – 8 s), and about 7%, 9%, 15% and 17% of molecules were detected compared with the first frame, respectively.

REFERENCE

(1) Ovesný, M.; Křížek, P.; Borkovec, J.; Švindrych, Z.; Hagen, G. M. ThunderSTORM: A Comprehensive ImageJ Plug-in for PALM and STORM Data Analysis and Super-Resolution Imaging. *Bioinformatics* **2014**, *30*, 2389–2390. https://doi.org/10.1093/bioinformatics/btu202.

(2) Dempsey, G. T.; Vaughan, J. C.; Chen, K. H.; Bates, M.; Zhuang, X. Evaluation of Fluorophores for Optimal Performance in Localization-Based Super-Resolution Imaging. *Nat. Methods* **2011**, *8*, 1027–1040. https://doi.org/10.1038/nmeth.1768.

(3) Rieger, B.; Stallinga, S. The Lateral and Axial Localization Uncertainty in Super-Resolution Light Microscopy. *ChemPhysChem* **2014**, *15*, 664–670. https://doi.org/10.1002/cphc.201300711.

(4) Nieuwenhuizen, R. P. J.; Lidke, K. A.; Bates, M.; Puig, D. L.; Grünwald, D.; Stallinga, S.; Rieger, B. Measuring Image Resolution in Optical Nanoscopy. *Nat. Methods* **2013**, *10*, 557–562. https://doi.org/10.1038/nmeth.2448.

(5) Culley, S.; Albrecht, D.; Jacobs, C.; Pereira, P. M.; Leterrier, C.; Mercer, J.; Henriques, R. Quantitative Mapping and Minimization of Super-Resolution Optical Imaging Artifacts. *Nat. Methods* **2018**, *15*, 263–266. https://doi.org/10.1038/nmeth.4605.

(6) Roberts, K. F.; Brue, C. R.; Preston, A.; Baxter, D.; Herzog, E.; Varelas, E.; Meade, T. J. Cobalt(III) Schiff Base Complexes Stabilize Non-Fibrillar Amyloid-β Aggregates with Reduced Toxicity. *J. Inorg. Biochem.* **2020**, *213*, 111265. https://doi.org/10.1016/j.jinorgbio.2020.111265.
